# Recent Progress in the Regeneration of Spinal Cord Injuries by Induced Pluripotent Stem Cells

**DOI:** 10.3390/ijms20153838

**Published:** 2019-08-06

**Authors:** Maria Csobonyeiova, Stefan Polak, Radoslav Zamborsky, Lubos Danisovic

**Affiliations:** 1 Institute of Histology and Embryology, Faculty of Medicine, Comenius University, Sasinkova 4, 811 08 Bratislava, Slovakia; 2 Department of Orthopaedics, Faculty of Medicine, Comenius University and National Institute of Children’s Diseases, Limbova 1, 833 40 Bratislava, Slovakia; 3 Institute of Medical Biology, Genetics and Clinical Genetics, Faculty of Medicine, Comenius University, Sasinkova 4, 811 08 Bratislava, Slovakia; 4 Regenmed Ltd., Medena 29, 811 01 Bratislava, Slovakia

**Keywords:** spinal cord injuries, induced pluripotent stem cells, differentiation, regeneration, disease modeling

## Abstract

Regeneration of injuries occurring in the central nervous system, particularly spinal cord injuries (SCIs), is extremely difficult. The complex pathological events following a SCI often restrict regeneration of nervous tissue at the injury site and frequently lead to irreversible loss of motor and sensory function. Neural stem/progenitor cells (NSCs/NPCs) possess neuroregenerative and neuroprotective features, and transplantation of such cells into the site of damaged tissue is a promising stem cell-based therapy for SCI. However, NSC/NPCs have mostly been induced from embryonic stem cells or fetal tissue, leading to ethical concerns. The pioneering work of Yamanaka and colleagues gave rise to the technology to induce pluripotent stem cells (iPSCs) from somatic cells, overcoming these ethical issues. The advent of iPSCs technology has meant significant progress in the therapy of neurodegenerative disease and nerve tissue damage. A number of published studies have described the successful differentiation of NSCs/NPCs from iPSCs and their subsequent engraftment into SCI animal models, followed by functional recovery of injury. The aim of this present review is to summarize various iPSC- NPCs differentiation methods, SCI modelling, and the current status of possible iPSC- NPCs- based therapy of SCI.

## 1. Introduction

Spinal cord injury (SCI) is a devastating trauma which causes long-lasting disability. SCI commonly results from dislocation or fracture of the spine in the cervical or thoracic region caused by various accidents [1]. The mechanical damage to the spinal cord right after the injury can result in the fast necrosis of nervous tissue associated with death of neurons and glial cells – that is, to primary (acute) injury seconds to minutes after SCI. Secondary injuries following disruption of the blood-spinal cord barrier and subsequent formation of free radicals and oxidative stress result in more neuronal and glial death. Other consequences include disintegration of myelin and demyelination. The activation of astrocytes, caused by infiltration of immune cells from the blood, results in reactive gliosis and subsequent formation of glial scars composed of extracellular matrix, glia and fibrotic cells. Secretion of chondroitin sulphate and other proteins inhibit axonal regrowth and prevent functional recovery of the spinal cord [2,3,4]. Moreover, loss of tissue volume may lead to the formation of microcysts, which subsequently merge together into large lesions lacking the extracellular substrate necessary for cell migration and growth. Current treatment options for SCI are mainly focused on stabilizing the spine, preventing the progression of secondary injuries, and controlling inflammation [5]. Currently, the only neuroprotective therapy approved for acute SCI is massive administration of methylprednisolone succinate (MPSS). However, the safety and efficacy of this treatment are disputable [6,7].

In the last decade, many reports have demonstrated significant recovery after early medical treatment of SCI; however, there is still no effective cure. Neuroregenerative strategies are focused on replacement of the damaged cells and axons by stimulating endogenous repair processes or by cell transplantation. The latter approach is considered a promising tool to replace damaged cells and promote neural protection and repair. The cell therapies offer to researchers and physicians the possibility to renew spinal cord function by inducing restoration of suitable conditions for the remyelination and growth of the axons with subsequent reconnection with new neurons [4,8,9]. The remyelination capacity of numerous cell types have been investigated including embryonic stem (ES) cells, adult neural stem/progenitor cells (NSCs/NPCs), olfactory ensheathing cells, and oligodendrocyte precursor cells (OPCs), as well as Schwann cells and bone marrow stromal cells. However, particularly significant promise has been demonstrated for NPCs isolated from fetal tissue or derived from ES cells. Nevertheless, as is well known, such use of embryonic tissue is highly restricted due to ethical concerns [10,11,12,13]. 

**Figure 1:**
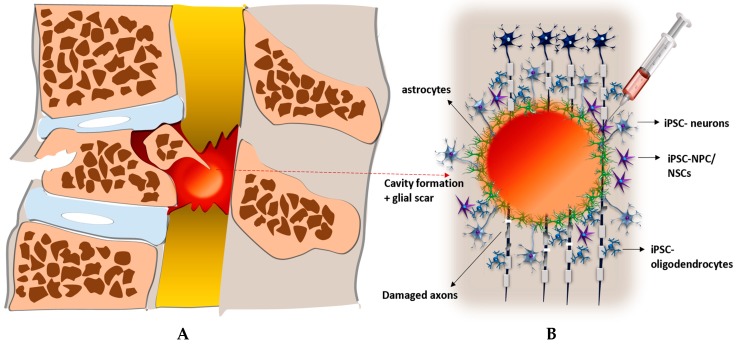
Transplantation of iPSCs in to the lesion after SCI and their targeting of ongoing pathological events. (**A**) Spinal cord after injury: formation of cystic cavity filled with glial scar, disruption of blood- spinal cord barrier. (**B**) Injection of iPSC-NPC into the site of lesion and their subsequent differentiation into the iPSC- neurons (replacing of dead neurons, axonal growth, synapses formation) and glial cells - iPSC- oligodendrocytes (remyelization of damaged axons), iPSC- astrocytes (reduction of inflammation, restoration of blood-spinal cord barrier).

The ground-breaking development of methods to generate induced pluripotent stem cells (iPSCs) from somatic cells has opened up new therapeutic opportunities for treatment of SCI. Shinya Yamanaka and his colleagues were the first to use retroviral introduction of key reprogramming factors (Oct4, Sox2, Klf4, c-Myc) into somatic cells to generate iPSCs. The use of iPSCs is particularly attractive, because it avoids the ethical and moral concerns that surround other stem cells. For application in the treatment of SCI, iPSCs can be differentiated into NPCs: neurons, oligodendrocytes, astrocytes, neural crest cells and mesenchymal stromal cells that can act to replace lost cells or provide environmental support (Figure 1). Several studies have shown that iPSC-derived cells can be safely transplanted into models of SCI and survive, integrate, and differentiate into desired phenotypes, as well as promote functional recovery. However, before such cells can be used in cell- based clinical therapy, several safety concerns still need to be resolved [1,14].

## 2. Differentiation of iPSCs into NPCs

It has been demonstrated that NPCs are among the best cell sources for providing functional recovery of SCI in animal models. For instance, Ružička et al. [15] compared three different cell sources (bone marrow-derived mesenchymal stem cells, NPCs derived from spinal fetal tissue, and iPSC-NPCs) in the treatment of balloon- induced SCI. In an animal model of locomotor recovery, the best results were obtained from the iPSC-NPCs, as assessed by the high rate of graft survival, increased axonal growth, and reduced glial scarring. 

**Figure 2:**
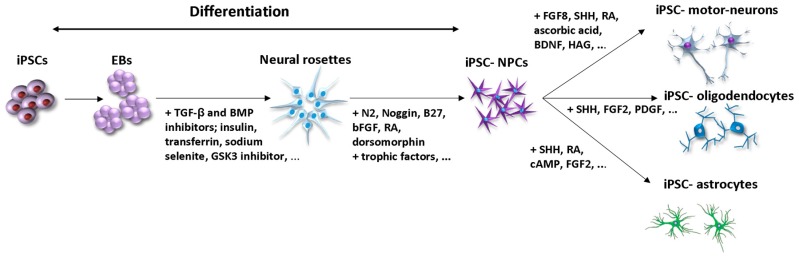
General steps of iPSC differentiation into neurons and glial cells through EBs and neural rosettes formation. The most used differentiation factors toward neural rosettes generation include TGF-β, BMP inhibitors, RA [18]; insulin, transferrin, sodium selenite [19] GSK3 inhibitor [20]. Further neural differentiation is supported by factors such as N2, bFGF, Noggin, B27, RA, trophic factors [18], dorsomorphine [21]. Lineage specific differentiation of iPSC- NPCs into motor neurons is controlled by FGF8, SHH [22], RA, ascorbic acid, BDNF, HAG [17]; into oligodendrocytes by SHH, FGF2, PDGF [20,23]; into astrocytes by SHH, RA, cAMP, FGF2 [24]

Differentiation methods initially used for ESCs have been successfully applied to iPSCs [16]. The induction of iPSCs into specific subtypes of neurons for particular replacement therapies has been promoted by alternating cell culture conditions and depends on the presence of specific microenvironmental factors and bioactive proteins (Table 1). During the last decade, iPSC–NPCs have been mostly generated by embryoid body (EB) formation and neural rosette isolation (Figure 2). Among methods for generation of iPSC- NPCs, generation via EB formation has been by far the most common. EBs are three-dimensional (3D) cell aggregates which can effectively initiate lineage-specific differentiation of iPSCs toward desired cell types. EBs can be differentiated into the NPCs by cultivation in medium containing TGF-β and BMP inhibitors together with Noggin and several small molecules, such as Y-27632, SB435142 and LDN193189, which are considered as enhancers of neuralization. Further neural differentiation can be guided by the specific neural induction medium supplemented by retinoic acid (RA), ascorbic acid, BDNF and SHH. Researchers by mentioned cultivations were able to differentiate NPCs into motor neurons expressing markers of limb-innervating lateral motor column motor neurons within 3 weeks [17]. The disadvantage of this protocol is its low efficiency; only a small percentage of cells are differentiated into NPCs, with other ectodermal and mesodermal cells lines also being induced. 

NPCs have also been generated from neural rosettes. The neural rosette is a distinctive cellular structure morphologically resembling the neural tube and represents a stage at which neural stem cells are highly proliferative with the capacity to differentiate into either neural or glial cell lineage under appropriate signals [19]. Neural rosettes are usually generated from ESCs/ iPSCs by cultivation in neurobasal medium supplemented by B27, N2 or Noggin. Neural progenitor cells can be further expanded and differentiated in the presence of fibroblast growth factor (FGF), or epidermal growth factor (EGF). By this method Lukovic et al. [19] differentiated NPCs into dopaminergic neurons and spinal motor neurons, as well as oligodendrocytes. For neural induction, authors used neural proliferation medium (NPM) containing factors, such as human recombinant bFGF, human insulin, ascorbic acid and RA and for oligodendrocytes induction was NPM further supplemented by triiodothyroidine and EGF. Authors demonstrated that simple proliferation medium, enriched with insulin, induced direct neural differentiation of approximately 98% of hiPCSs into neural progenitors. Moreover, used proliferation medium was animal-free containing human extracellular matrix components, which makes this method more appropriate for future clinical applications. 

Another differentiation method based on neural rosettes induction was published by Nutt et al. [18]. Authors cultivated neural rosettes derived from iPSCs in DMEM F12 medium supplemented by N2, bFGF, and sonic hedgehog with later addition of cAMP, T3, PDGF, IGaF-1, and neurotrophin-3. RA as the caudalizing factor, was also added to induce expression of HoxB4, the hindbrain/spinal cord specific transcription factor. After 45 days, aggregates of TuJ1- positive neurons, GFAP- positive astrocytes and less number of GSTpi- positive oligodendrocytes were observed. The iPSC-NPCs were injected (100 000 cells) into the dorsolateral funiculus of rats four weeks after induced cervical SCI. The iPSC-NPCs were able to integrate into and generate multiple neural cell lineages in this transplanted environment, although recovery of function in this chronic SCI model was limited. The poor recovery outcome emphasizes the need to focus on development of more precise and effective protocols, especially for treatment of chronic SCI.

Chandrasekaran et al. [25] compared efficacy of monolayer based neural induction method with spheroid (3D) based induction through dual inhibition of the SMAD signalling pathway. Neural rosettes, expressing SOX1, NESTIN and PAX6, were induced by SB431542 and Noggin and expressed similar morphology in both ways of induction. Further cultivation of neural rosettes in neural maintenance medium containing EGF and bFGF leaded to generation of motor neurons. However, there were detected slight differences in axonal growth and maturity of neurons in favour of 3D induction method. Nevertheless, the spheroid generation is associated with problems involving proper control of their size and shape, as well as with number of mature cells forming spheroids. 

It’s generally known, that protocols based on the methods described above are time-consuming and result in insufficient amounts of differentiated cells [21]. A rapid single-step induction of iPSCs into neurons was published by Zhang et al. [26]. This method is based on forced overexpression of neurogenine-2 or NeuroD1 with a lentiviral vector. Surprisingly, the conversion of iPSCs into NP cells was achieved in less than one week and mature mostly excitatory cortical neurons were observed in two weeks. The neurons were able to exhibit spontaneous synaptic activity with short term plasticity. However, the use of lentiviral vector is associated with potential safety risks; thus, these differentiated neurons are more suitable for drug screening and modelling of neurodegenerative diseases than for clinical treatment. 

Badja et al. [22] published an iPSC-NPCs differentiation protocol based on a feeder-free method and use of chemically defined medium, without need for EBs formation. Four days after induction in the presence of dimethyl sulfoxide, expression of the neural stem cell marker NESTIN was detected. Further differentiation was achieved by cultivation on laminin-coated dishes in neural differentiation medium without bFGF and EGF. The final differentiation into dopaminergic neurons was induced in presence of specific ctytokines, such as FGF8 and SHH. The resulting terminal neurons expressed voltage-gated receptors for GABA, glycine, and acetylcholine. The advantages of this method include relatively short time of neural differentiation process (20–40 days), large scale production of mature neurons and its cost- effectiveness. However, neural differentiation is a multistep process, which can result in a heterogeneous cell population, causing difficulties in obtaining cells at the same mature stage [27].

A new method for obtaining neural stem cells (NSCs) from iPSCs was developed by Choi et al. [28]. This novel approach of generation iPSC- NSCs is based on chimera formation in vivo. Briefly, the iPSCs were aggregated with morulas and formed chimeric blastocysts, which were transferred into pseudo-pregnant mother resulting in generation of chimeric embryos. Subsequently, the brain tissue was separated and trypsinized to release single cells. Cells were cultured in neurosphere medium and after 10 days, the populations of iPSC-derived NSCs, expressing NESTIN and Sox2, were observed. The chimeric iPSC-derived NSCs displayed similar features (morphology, gene expression) as fetal brain-derived NSCs. The advantage of this method is that NSCs can be efficiently isolated from chimeric brain tissue, purified, and cultured in vitro. Nevertheless, the use of a chimera formation method for therapeutic application in humans could raise ethical concerns. 

## 3. Advances in Scaffold Construction

An important component in neural differentiation technique, besides composition of induction medium, is the use of a suitable scaffold. Currently there are several protocols, based on traditional two-dimensional systems (2D), such as adherent monolayers and stromal feeder layers, or three-dimensional systems (3D), such as hydrogels, which provide higher differentiation efficiency of iPSCs for large-scale NPCs generation. The 3D systems represents much more advanced technique because they are constructed to mimic natural microenvironment of the nervous tissue. It was proved that 3D hydrogel systems composed of electrospun fibers can partially induce the neural differentiation. Such tissue engineered nanofiber scaffolds can be constructed from various biocompatible polymers. 

The cell growth, survival and proliferation of NSCs seeded on 3D electrospun poly- (lactide-co-glycolide)/polyethylene glycol (PLGA-PEG) nanofiber scaffolds was investigated by Liu et al. [37]. NSCs were reprogrammed directly from mouse fibroblasts and cultivated in the absence of EGF and FGF, but in presence of FBS and RA. Cultivation leaded to Tuj1-positive neurons and MAP2-positive neurons. Moreover, part of iNSCs differentiated into GFAP-positive astrocytes and MBP-positive oligodendrocytes. The regenerative potential of induced cells was examined on in vivo rat model with transected spinal cord. The neural differentiation of seeded cells was observed within two to eight weeks post transplantation. The double immunostaining method showed successful integration of implanted scaffold inside the lesion cavity and presence of differentiated neurons and glial cells in surroundings of the lesion. These results represent promising way for scaffold construction with long-term survival capacity, which can be suitable for SCI treatment. 

Mohtaram et al. [38] used an innovative melt electrospinning method without the need to use of cytotoxic solvents. The researcher group generated poly(ε-caprolactone) (PCL) scaffolds with loop mesh and biaxial aligned microscale topographies enriched with RA. Constructed scaffolds were subsequently loaded with neural aggregates composed of 4000–5000 iPSC-NPCs. After 12 days, seeded cells were able to adhere to scaffolds and migrate along fibers. Successful differentiation into the neurons was proved by their positivity to neuronal marker Tuj1. Moreover, obvious neurite outgrowth was also present. Taken together, composition of such bimodal scaffolds is supporting neural differentiation by formation of appropriate chemical and physical microenviroment for seeded cells. 

The electrospinning technique to synthesize a biocompatible and biodegradable novel poly- caprolactone (PCL) scaffold was used also by Zhou et al. [39]. The PCL scaffold was coated with Schwann cells and iPSC-NPCs and transplanted into in vivo SCI rat model. iPSC-NPCs were generated by EBs method involving their cultivation in EBs medium containing dorsomorphin and neurobasal medium. After obtaining EBs, the medium was changed to NSCs medium enriched with EGF and FGF. Eight weeks after transplantation, the more significant and faster locomotor recovery was observed in comparison with the group treated only by PCL scaffold without cell coating. However, the authors did not examined transfected cells for their possible tumorigenicity. 

The new look at 3D system composition was recently published by Fan et al. [40]. The authors developed 3D gelatine methacrylate (GelMA) hydrogel as a scaffold for iPSC-NPCs. Cells were differentiated toward neural lineage through EBs formation and their subsequent cultivation in NSC differentiation medium containing RA. Obtained iPSC-NPCs expressed neural progenitor marker, such as nestin and PAX6. After their transplantation into a SCI mouse model, robust neural differentiation was observed, together with notable cavity reduction and the suppression of inflammation. Generally, despite the positive features of hydrogels (content similar to extracellular matrix; non-invasive injectable way of transfection), the process of construction is often difficult depending on specific construction of the molecules [41]. 

All the studies mentioned above prove the significant impact of scaffold composition in the processes of neural differentiation, graft survival and final SCI regeneration. 

## 4. SCI Modeling and Therapy with iPSC-Derived NPCs

For understanding of the pathologic processes underlying SCI injury and for development of treatment strategies, the use of animal models is inevitably necessary. Rodents are the most frequently used animal model due to their ease of handling and monitoring of physiological functions. However, rodent models can display spontaneous recovery after even massive SCI, which can be undesirable for final evaluations. Other animal models include dogs and non-human primates [42]. Because NPCs are one of the most promising cell types for treatment of SCI, several research groups have done pioneering studies examining the transplantation of iPSC–derived NPCs for treatment of SCI, with hopeful results [29,31,32]. 

Qin et al. [43] published a meta-analysis of randomised control pre-clinical trials regarding effectivity of SCI repair after iPSC-NPCs injection. Data were obtained from 79 studies including 212 rat models. According to this analysis, iPSC- NPCs transplantation considerably promotes locomotor recovery. The authors concluded that iPSC- NPCs are able to initiate microvascular and nerve regeneration, reduce inflammation and oxidative stress, and enhance axonal growth. According to the meta-analysis, the most effective method of iPSC- NPCs transplantation is intraspinal implantation during the subacute phase (2–4 weeks after injury) of injury. However, this method may cause unwanted additional damage of the spinal cord. Moreover, there are some major difficulties with autograft-based cell therapy; for instance, it takes about a few months to establish iPSC lines; it takes three months to induce these iPSCs into NPCs in vitro, and an additional year would be required for quality control, including evaluation of cytogenetic instability and activation of telomerase activity or selected genes associated with cell cycle regulation and apoptosis induction leading to malignant transformation [44,45,46]. 

The therapeutic potential of human iPSC-derived neurospheres (hiPSC-NS) in treatment of SCI was studied by Nori et al. [29]. Human hiPSC-NS were grafted into a mouse model of SCI and were able to survive and differentiate into the neurons, and glial cells within the lesion. The synapse formation between injected human hiPSC-NS–derived neurons and host neurons was demonstrated. Moreover, the new differentiated neurons expressed neurotrophic factors. During the long-term observation period (112 days), the process of angiogenesis and axonal regrowth was also detected without any tumor formation. This pre-clinical work confirmed that hiPSCs represents a suitable source of neural cells which can be used for SCI therapy in very close future.

Tsuji et al. [30] succeeded in restoring motor function by transplanting iPSC–NPCs into a mouse model of SCI, and reported that when “good iPSCs”– NPCs which had been pre-evaluated as non-tumorgenic by transplantation into the brains of immunocompromised mice – were used for transplantation, motor function was restored without tumor development over an observed long period of time. This study is confirming an importance of cell graft safety pre-examination prior to transplantation in order to avoid tumor formation.

In 2013, Nutt et al. [18] first examined the pre-clinical potential of human iPSC- derived NPCs regarding regeneration of nerve tissue after chronic SCI. Authors generated and injected desired specific type of iPSC- NPCs into the model of chronic cervical SCI. Although the engraftment and neural differentiation potential of iPSC- NPCs was significant, the notable progress in forelimb function was not observed. This data indicates that there is still a need to focus on specific hiPSCs derivatives or co-therapies that will restore function in the early chronic injury setting. 

The axonal growth of iPSC- NSCs in relation to inhibitory environment of the adult spinal cord was investigated by Lu et al. [33]. Authors reprogrammed dermal fibroblasts from 86-year-old human into iPSCs and differentiated them into NSCs. The mature neurons and glia derived from iPSC- NSCs were observed three month after their transplantation and engraftment into the immunodeficient rat model of SCI (C5 lateral hemisection). Moreover, enormous axonal growth took place at the site of injury and grown further over the length of rat central nervous system. These axons were able to form new synapses with host’s nerve fibres. Interestingly, authors proved that despite the high age of donor, the iPSC-NSCs retain their high plasticity overcoming inhibitory effect on the axonal growth of adult SCI. Nevertheless, recipient oligodendrocytes were not able to myelinate graft-derived human axons. 

Romanyuk et al. [35] examined functional recovery in rat SCI by several approaches, such as the Basso, Beattie and Bresnahan (BBB) locomotor scale method, the beam walking rotarod, and the plantar test. Human iPSC-NPCs were injected into the spinal cord lesion one week after SCI. Functional recovery of hind limb motor function was observed already in the second week after iPSC-NPCs transplantations. Grafted cells actively took part in axonal regrowth and trophic support in the site of injured tissue. Moreover, through the period of observation, no tumor formation was detected. Final differentiation of iPSC-NPCs into the several types of neurons (interneurons, dopaminergic neurons, serotoninergic neurons) was observed at week 17. According results, human iPSC-NPCs display high neurotrophic activity resulting in eminent locomotor recovery after SCI. 

Suzuki et al. [47] used the clip-contusion mice model of cervical SCI to investigate if the initial treatment of chronic SCI with intrathecal chondroitinase ABC (ChABC) can improve regenerative effectiveness of transplanted iPSC-NPCs. Authors delivered ChABC into the site of injury and subsequently injected iPSC-NPCs after the seven weeks from SCI. Results proved reduction of lesion site, massive neural differentiation and synapses formation suggesting possible treatment option of ChABC in chronic SCI model. Group of Okubo et al. [48] examined the efficacy of gamma-secretase inhibitor (GSI) treated iPSC- NSC/NPCs in NOD/SCID mouse model of chronic SCI. The GSI is known to be inhibitor of Notch signaling, which can induce neural differentiation. The GSI treated iPSC- NSC/NPCs were injected into the damaged lesion at day 42. After transplantation, the cells survived without any tumor formation for 3 months. Performed quantitative analyses showed significant remyelination and according immunostaining method, the axonal regrowth was detected too. Finally, these events led to locomotor function improvement, suggesting the positive influence of GSI on SCI regeneration.

Recently it was also demonstrated that oligodendrocyte progenitors (OPs) can be derived from iPSCs and survive and differentiate into mature oligodendrocytes after early transplantation into the injured spinal cord. Animals that received transplantation of iPSC-OPs 24 h after injury showed a significant increase in myelinated axons. Furthermore, a 5-fold reduction of the lesion site together with minimization of the glial scar was observed. Mentioned events are considered to be essential for regeneration of damaged nervous tissue and recovery from SCI. Thus, iPSC- OPs along with iPSC- neurons represent more effective way for SCI treatment than iPSC- neurons themselves [19]. Similarly, Kawabata et al. [20] differentiated OPs from iPSC- NPCs and injected them into NOD/SCID mice after SCI. Approximately 40% of the iPSC-OPs differentiated into mature oligodendrocytes which were able to remyelinate the injured axons. Moreover, axonal regrowth and formation of synapses were detected. Yang et al. [23] investigated the relation between miRNA expression and the positive effect of transplanted iPSC- OPs in rat SCI model. Authors identified the most upregulated (miR-375-3p and miR-1-3p) and downregulated miRNA (miR-363-3p, miR-449a-5p and miR-3074) by quantitative PCR. According to the bioinformatics analyses authors suggested that these results indicate that iPSC- OPs may improve SCI recovery. Astrocytes are another type of glial cell which can be differentiated from iPSCs. Hayashi et al. [24] differentiated iPSC- derived astrocytes by the neural stem sphere method and injected them at days 3 and 7 into the T9-10 SCI rodent model. Following transplantation, the authors observed and evaluated cell activity during an 8-week period. However, no significant locomotor recovery was detected; furthermore, transplantation of iPSC-derived astrocytes caused an increase of sensitivity to mechanical stimuli. 

## 5. Important Considerations before Clinical Application of iPSCs-NPCs in SCI

Successful recoveries of SCI in mostly rodent animal models have brought new hope for SCI patient treatment. However, therapies effective in rodent models may fail in primates due to several factors, such as differences in intrinsic biology, small effect size, safety and tolerability issues, and inappropriate adjustment of methods to humans. Currently, investigation of effectiveness of iPSCs-NPC treatments in primate SCI models is seen as a key step toward successful clinical trials [49,50]. In 2012, Kobayshi et al. [32] reported the use of a non-human primate model, representing a missing link between rodent and human studies. In this preclinical study, researchers used an adult common marmoset (*Callithrix jacchus*) model of SCI to evaluate the regenerative effect of transplanted hiPSC-NSCs. Axonal regrowth and angiogenesis without tumor formation were detected within 12 weeks of observation. Moreover, behavioural tests (cage climbing and the bar grip test) demonstrated the ability of hiPSC-NSCs to promote long-term functional recovery. Recently, Rosenzweig et al. [49] published work, in which authors successfully restored cervical (C7) SCI in non-human primate model (*Macaca mulatta*) by implantation of NPCs into the lesion cavity two weeks after injury. During 9 months of observation period, the grafts underwent maturation, together with massive axonal extension through monkey white matter and subsequent synapse formation in gray matter. Moreover, monkeys with surviving grafts remarkably improved their overall motor function.

According to the above-mentioned studies, iPSCs technology has an enormous therapeutic potential, offering new opportunities for cell transplantation therapy in SCI. However, there are still several major challenges that need to be resolved during the pre-clinical phase. Among them, the most pressing issue is efficacy and safety of the whole process, which starts with harvesting of suitable somatic cells from donors, followed by their viral-free reprogramming into the iPSCs; their complete differentiation into neural progenitor cells/mature neurons/ glial cells; purification, and finally, their transplantation into the site of injury. The safety risks are mostly related to genetic and epigenetic abnormalities, tumorigenicity, and immunogenicity of transplanted cells. Regarding the tumorigenicity of iPSCs as related to their tissue of origin, Miura et al. [51] examined 36 lines of iPSCs. The authors found that the most suitable iPSC lines for NPCs differentiation were those obtained from mouse embryonal fibroblasts and gastric epithelial cells. These iPSC-NSCs showed a stable differentiated population in neurospheres in vitro and almost no teratoma formation after transplantation into a rodent brain. On the other hand, iPSC lines obtained from adult tail-tip fibroblasts displayed residual Nanog+ undifferentiated cells, resulting in a higher count of tumor cells. The results of this study demonstrated that the selection of proper tissue source is an essential step toward generation of safe iPSCs. Another approach to reducing the risk of tumor formation from iPSC-NPCs has been suggested by the work of Okubo et al., who found that pre-treatment of hiPSC-NPCs before transplantation with a y-secretase inhibitor (GSI) led to more neuronal cells with less proliferative capacity, resulting in a reduction of tumor-initiating cells [36]. As a “fail-safe” system to address tumors that do form after iPSC-NPC transplantation, Itakura et al. [34] showed that such tumors in a rodent model could be ablated by cessation of immunosuppressants. To increase the efficacy of the SCI repair process, Kitamura et al. [52] introduced exogenous hepatocyte growth factor (HGF) into the injured rat spinal cord via an HGF-expressing viral vector. This resulted in a significant increase of grafted iPSC-NPCs differentiation into neurons and oligodendrocytes and their survival by prevention of apoptosis. Moreover, HGF supported angiogenesis and axonal re-growth, and other studies have shown that exogenously administered HGF reduces glial scar formation. They also extended their initial rodent experiments to intrathecal administration of recombinant human HGF (rhHGF) into adult marmosets after cervical SCI at the C5 level. The rhHGF was injected for four weeks and led to eminent recovery of upper limbs in all five marmosets. Based on these results, the authors suggested that combination therapy involving HGF administration together with iPSC-NPCs transplantation may considerably enhance regenerative therapy after SCI. 

Another substantial aspect to be considered is the effect of the post-SCI microenvironment on hiPSC-NSCs. According to a study by López-Serrano et al. [53], the pro-inflammatory tissue microenvironment may induce proliferation of transplanted cells. Therefore, it is necessary to study graft-host interactions in the SCI microenvironment in order to develop the most suitable patient-specific therapy adjustments. 

Surprisingly, SCI regeneration by iPSCs technology is much closer to clinical use today than it has ever been before. In February 2019, the Health Ministry of Japan approved the world’s first clinical trial to test the therapeutic potential of hiPSC-NPCs in four patients who suffered acute SCI. The trial will be conducted by Hideyuki Okano, a professor at Keio University, and his team. Researchers are planning to inject hiPSC-NPCs within two to four weeks of a patient’s injury and to observe the efficacy and safety of the cells for about a year, while the patients undergo rehabilitation. 

## 6. Conclusions 

Undoubtedly, the clinical use of iPSCs for SCI regeneration is right around the corner. Stem cell transplantation is an attractive therapeutic option as iPSC-NPCs grafts support multiple mechanisms leading to recovery from SCI. One of the major advantages is their ability to overcome the ethical issues and to avoid the immune rejection, because of their somatic origin and patient- specificity. However, despite the rapid advance of the iPSCs technology, there still remain few, but essential hurdles to overcome, such as possible risk of tumor formation. The scientific effort is now focused on the development of safer and more efficient reprogramming technique by improving transgene-free and non-viral methods and on refinement of desired cell type differentiation and purification.

## Figures and Tables

**Table 1:** Overview of protocols for iPSC- NPC/NSCs induction.

Animal Model	Lesion Type	Lesion Site	Starting Cell Type	Obtained Cells After Neural Induction	Differentiated Cell Types for TP	Timing of TP After SCI	Outcome after TP and Time of Recovery	References
**C57BL/6N adult mouse**	Contusion injury by IH impactor	T10	Mouse iPSCs	iPSC- derived neurospheres	iPSC-NPCs	9 days	Functional recovery – 21 d.; inhibition of astrogliosis, no teratoma formation	[29]
**NOD/SCID mouse**	Contusion injury by IH impactor	T10	Mouse iPSCs	iPSC- derived neurospheres	iPSC-NPCs	9 days	Functional recovery – 42 d.; no tumor formation	[30]
**NOD/SCID mouse**	Contusion injury by IH impactor	T9-T10	Mouse iPSCs	iPSC- derived neurospheres	iPSC- astrocytes	3–7 days	No functional recovery; increase of sensitivity to mechanical stimuli	[24]
**NOD/SCID mouse**	Contusion injury	T10	Human iPSCs	iPSC- derived neurospheres	hiPSC-NPCs	7 days	Functional recovery – 8 w., graft survival with incomplete filling of lesions	[31]
**Common marmoset**	Contusion injury by weight-drop device	C5	Human iPSCs	iPSC- derived neurospheres	hiPSC- NSCs	9 days	Functional recovery – 56 d., angiogenesis, remyelinization; no tumor formation	[32]
**Long- Evans rat**	Hemi-contusion by Ohio State Injury Device	C4	Human iPSCs	iPSC- derived neural tube rosettes	hiPSCs - NPCs; iPSC-OPs	4 weeks	No functional improvement; graft survival with incomplete filling of lesion	[18]
**Athymic nude rat**	Lateral hemi-section	C5	Human iPSCs	iPSC- derived neural tube rosettes	hiPSC-NSCs	2 weeks	No functional recovery; robust extension of axons without myelination;	[33]
**BALB/cA mouse**	Laminectomy	T10	Human iPSCs	iPSC- derived neurospheres	Tumorigenic hiPSC-NSC/NPCs	daily (28 days)	Massive rejection of hiPSC-NSC/NPC – based tumors cause by cessation of immunosuppressants	[34]
**Wistar rats**	Balloon induced-compression	T8-T9	Human iPSCs	iPSC- derived EBs	iPSC-NPCs	7 days	Functional recovery – 14 d.; differentiated neurons, oligodendrocytes, astrocytes; axonal regrowth	[35]
**NOD/SCID mouse**	Contusion injury	T10	Human iPSCs	iPSC- derived neurospheres	OPs- derived from hiPSC-NPCs	9 days	Improvement of functional recovery- 35 d.; injured axons remyelination; no tumor formation	[20]
**Wistar rats**	Balloon induced-compression	T10	Human iPSCs	iPSC- derived neurospheres	iPSC-NPCs	7 days	Functional recovery – 8 w.; reduced astrogliosis; decrease inflammation	[15]
**Wistar rats**	Contusion injury	T9-T11	Mouse iPSCs	iPSC- derived EBs	OPs- derived from iPSC-NPCs	7 days	Possible promotion of functional recovery based on the results of a miRNA assay – 7 d.	[23]
**NOD/SCID mouse**	Contusion injury	T10	Human iPSCs	iPSC- derived EBs	hiPSC-NSC/NPCs	9 days	Improvement of locomotor function; axonal regrowth and remyelination – 42 d.; no tumor formation	[36]

IH: Infinite Horizon impactor.
